# Mitigating human–wildlife conflict and monitoring endangered tigers using a real-time camera-based alert system

**DOI:** 10.1093/biosci/biad076

**Published:** 2023-09-14

**Authors:** Jeremy S Dertien, Hrishita Negi, Eric Dinerstein, Ramesh Krishnamurthy, Himmat Singh Negi, Rajesh Gopal, Steve Gulick, Sanjay Kumar Pathak, Mohnish Kapoor, Piyush Yadav, Mijail Benitez, Miguel Ferreira, A J Wijnveen, Andy T L Lee, Brett Wright, Robert F Baldwin

**Affiliations:** Clemson University, Clemson, South Carolina, United States; Clemson University, Clemson, South Carolina, United States; RESOLVE, Washington, DC, United States; Wildlife Institute of India, in Dehradun, Uttarakhand, India; Global Tiger Forum, New Delhi, India; Global Tiger Forum, New Delhi, India; RESOLVE, Washington, DC, United States; Dudhwa Tiger Reserve, Uttar Pradesh, India; Global Tiger Forum, New Delhi, India; RESOLVE, Washington, DC, United States; CVEDIA Ltd, in Reading, Berkshire, United Kingdom; CVEDIA Ltd, in Reading, Berkshire, United Kingdom; CVEDIA Ltd, in Reading, Berkshire, United Kingdom; RESOLVE, Washington, DC, United States; Tigers United University Consortium, Clemson University, in Clemson, South Carolina, United States; Clemson University, Clemson, South Carolina, United States

**Keywords:** embedded-AI camera-alert systems, tigers, human–wildlife conflict, poaching, endangered species

## Abstract

The recovery of wild tigers in India and Nepal is a remarkable conservation achievement, but it sets the stage for increased human–wildlife conflict where parks are limited in size and where tigers reside outside reserves. We deployed an innovative technology, the TrailGuard AI camera-alert system, which runs on-the-edge artificial intelligence algorithms to detect tigers and poachers and transmit real-time images to designated authorities responsible for managing prominent tiger landscapes in India. We successfully captured and transmitted the first images of tigers using cameras with embedded AI and detected poachers. Notifications of tiger images were received in real time, approximately 30 seconds from camera trigger to appearing in a smart phone app. We review use cases of this AI-based real-time alert system for managers and local communities and suggest how the system could help monitor tigers and other endangered species, detect poaching, and provide early warnings for human–wildlife conflict.

The rebounding of wild tiger (*Panthera tigris*) populations in India and Nepal has been one of the great conservation success stories of the first quarter of the twenty-first century. Although tigers are still endangered, from a rapidly declining global tiger population 20 years ago, global estimates have increased to approximately 4500 individuals (Dinerstein et al. 2007, Goodrich et al. 2022). Tigers have recovered in large part because of the efforts of governments in tiger-range countries and nonprofit and nongovernmental organizations. The rebound was accelerated by the St. Petersburg Declaration on Tiger Conservation, crafted in 2010 to conserve vital tiger habitat, abate rampant poaching, and target a doubling of the wild population by 2022 (Global Tiger Initiative Secretariat 2011, Global Tiger Forum 2019).

Simultaneously, although tigers’ numbers have increased, human populations have expanded in the vicinity of tiger reserves, leading to inevitable encounters and conflict. Human populations surrounding nearly all internationally recognized Tiger conservation landscapes (TCLs; Dinerstein et al. 2007) have also grown by over 19.5 million people from 2000 to 2020 (figure 1a, supplemental table S1). The expansion of human development into tiger habitat exacerbates the threats of retaliatory killing, poaching, prey depletion, and habitat degradation for tigers and injury or death for livestock and people (Miller et al. 2016, Jhala et al. 2021). The renewed vision for tiger conservation, following the second Tiger Summit in Vladivostok, Russia, in 2022, demonstrated that there is a continued commitment to a landscape-scale management approach that ensures the sustained population growth of this globally imperiled species while sustaining human livelihoods near tiger habitat. These twin goals will require further expansion of protected areas within the world's TCLs and the establishment of corridors linking protected areas that, on their own, remain too small to sustain long-term viable tiger populations without allowing for tiger dispersal and permitting gene flow among reserves (Dinerstein et al. 2006, Wikramanayake et al. 2011, Joshi et al. 2016).

**Figure 1.**
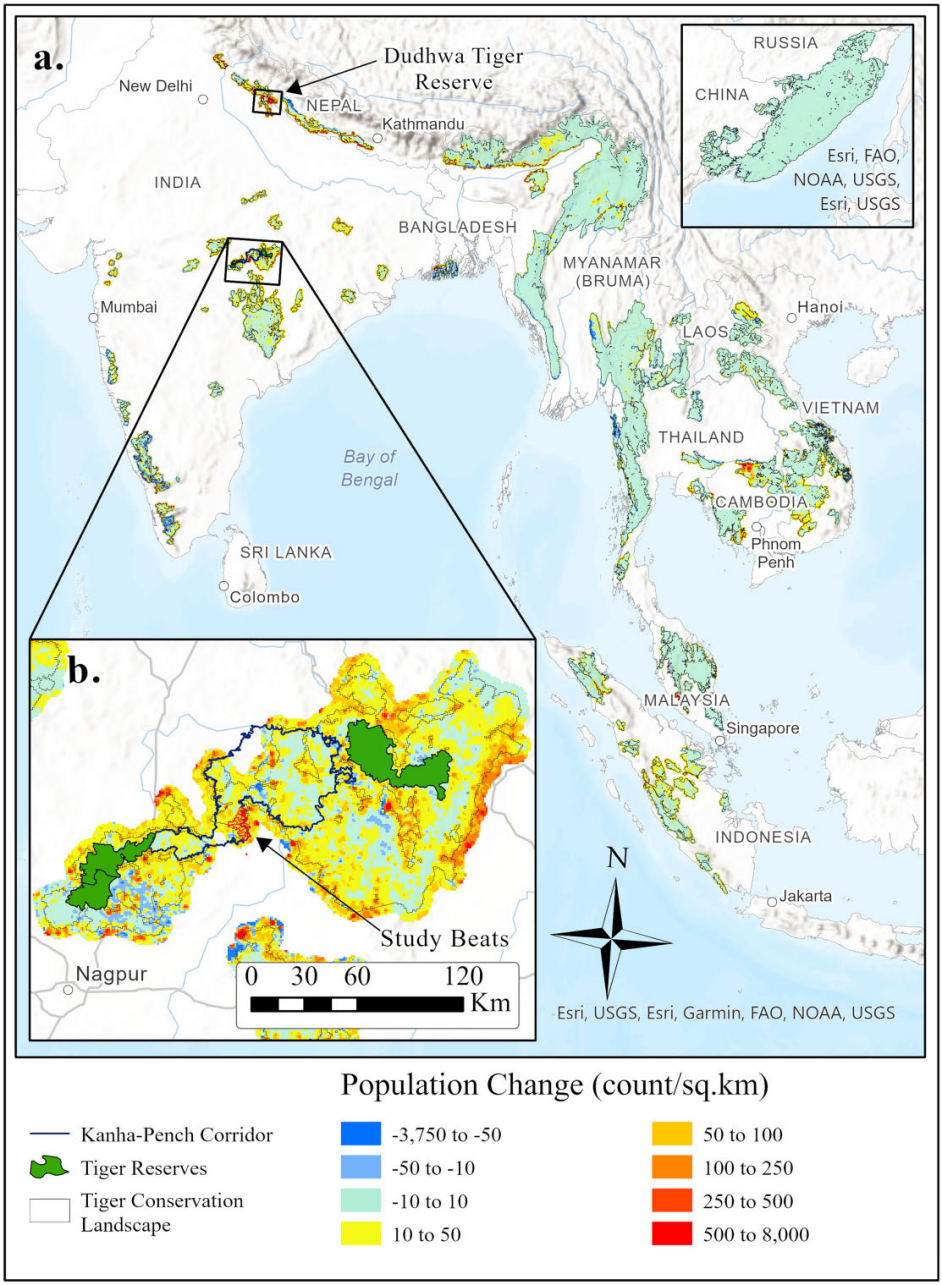
(a) The human population surrounding the world's tiger conservation landscapes (TCLs) increased by over 19.5 million people between the years 2000 and 2020. These population increases are especially acute for TCLs in northern and central India, Cambodia, and Sumatra. (b) Our initial deployment of the TrailGuard AI camera-based alert system focused on central India in the partially protected forest corridor between Kanha Tiger Reserve in the northeast and Pench Tiger Reserve to the southwest. An additional deployment occurred in northern India within Dudhwa Tiger Reserve. Source: The population data were generated using global population estimates from the WorldPop Hub (WorldPop 2018). The data were clipped to include the area within a 5-kilometer buffer around all TCLs to capture population growth in close proximity to potential tiger habitat. The inset map of the Russian Far East TCL is not at the same scale as the overall map.

In addition, there is increasing recognition of the permanent occupancy of tigers outside tiger reserves. In India, which contains approximately 66% of the world's wild tiger population, approximately 35% of this increasing population permanently resides outside designated tiger reserves (Jhala et al. 2019). A remarkable finding from the most recent survey is that the number of wild tigers within India but existing outside a protected tiger reserve is approaching the number of tigers in all other tiger range countries combined (table 1). The dispersal requirements of the species dictate that individual tigers will increasingly use the interstitial landscapes outside protected areas, therefore occupying habitat highly disturbed by humans (Sunquist and Sunquist 2002). A complicating factor is that the tiger population is likely increasing in many areas in which forest rangers and guards have less training and fewer resources to manage a permanent tiger presence than within designated tiger reserves (Milda et al. 2020). The continued recovery of the tiger population, the dispersal requirements of the species, and higher human densities surrounding most TCLs (figure 1a), increases the risk of poaching and human–tiger conflict.

**Table 1.** Comparison of tiger estimates across different regions.

	Population	Estimate	World
Region	estimate	range	population percentage
World	4485	3726–5578	100
India	2967	2603–3346	66.2
Other tiger range countries	1518	1033–2232	33.8
Outside India's tiger reserves^a^	1044	–	23.2
Central India^b^	883	753–1026	19.7
Kanha–Pench Landscape	308	252–370	6.9

*Note:* India contains the majority of the world's wild tigers, and of those individuals, over one-third use habitat outside a protected tiger reserve. The tiger metapopulation in Central India is the largest in the world with the Kanha–Pench population block being the largest in that region. The population block acts as an important tiger source population and is a larger tiger population than all but three tiger range countries (Indonesia, Russia, and Nepal). *Source:* The estimates were taken from Goodrich and colleagues (2022) and Jhala and colleagues (2019), and the data were collected from 2014 to 2020.

^a^Estimate of resident tigers in India outside of a designated tiger reserve (Jhala et al. 2021).

^b^Central India, for these purposes, includes the states of Madhya Pradesh, Chhattisgarh, Maharashtra, and Telangana. Almost all of Maharashtra's tiger population resides in the eastern portion of the state.

Human–tiger conflict can take several forms, including the killing of livestock and attacks on people by tigers (Dhanwatey et al. 2013, Karanth et al. 2013) and the retaliatory killing of tigers by angered community members (Inskip and Zimmermann 2009). The resulting human and tiger casualties could reverse wild tiger population gains while further threatening support for conservation among community members in and around TCLs (Goodrich 2010, Letro and Fischer 2020). With forest protection staff spread so thinly across large landscapes (Appleton et al. 2022), new technology that can provide real-time data notifications for conservation personnel could be a massive aid in preventing poaching or conflict before it occurs or afterward for quickly identifying poaching suspects (Hahn et al. 2022). Forms of early detection solutions for remote monitoring of vital forested tiger habitat have existed (Joshi et al. 2016) and are rapidly being improved on (Global Forest Watch 2022), but on-the-ground early-warning solutions for protecting wildlife have been slower in development (Dinerstein et al. 2006). A solution that could provide real-time information on tiger and poacher activity across a landscape would constitute a foundational shift in the ability for wildlife managers and community members to abate conflict and intercede quickly in poaching to better protect one of the world's most imperiled species.

In addition, because shared landscapes outside of protected areas in India currently represent a vital part of tiger and other large carnivores’ remaining habitat, the viability of these populations may increasingly rely on managing for coexistence with humans, especially where protected area expansion is unlikely (Inskip and Zimmermann 2009, Carter and Linnell 2016). Therefore, conservation solutions that can reduce communications barriers between conservation managers and local communities to stop poaching and prevent human–wildlife conflict are among the most urgent needs in tiger conservation and wildlife management globally.

## From camera traps to AI-embedded camera-based alert systems

Camera traps have become a vital tool for biologists and natural resource practitioners in the conservation of wildlife species (Rich et al. 2016, Jhala et al. 2019, Kays et al. 2022). This application is especially true over the last two decades for monitoring and estimating the demographics of tigers and other felid species (Karanth et al. 2017). Although traditional camera traps have allowed for answering a plethora of questions on the abundance, presence, and temporal overlap of species, there are numerous issues that can come about during their usage (Glover-Kapfer et al. 2019). These can include mass collection of false triggers, the costs of gathering and processing data, and stolen cameras, all of which reduce the effectiveness of cameras as a tool for conservation. Until recently, camera traps have lacked the real-time notification capabilities and practicality necessary for prevention of poaching and human–wildlife conflict.

Technology is newly emerging to connect authorities with rapidly obtained management data that could be used to resolve conflict. Methods to use AI to identify species combined with communications networks to detect endangered species and alert managers have been tested such as for elephants in Gabon (Whytock et al. 2023). But, as of yet, no such system has used AI on-the-edge computing to filter data notifications to just species of interest nor been tested for wild tigers or had its capabilities tested for a large predator in peopled mosaics such as outside tiger reserves. TrailGuard AI (Nightjar, Washington, DC, in the United States) is an advanced camera-based alert system that provides real-time data on wildlife and human presence. The camera portion uses embedded AI via an advanced computer vision chip (two versions, from Intel; see supplemental file S1) to autonomously identify and filter detections of wildlife species of interest, as well as humans. The AI is trained using a combination of three-dimensional species renderings (CVEDIA Ltd., Reading, Berkshire, United Kingdom) and validated using camera trap photos in order to improve the AI's predictive ability in identifying unique species and providing situational awareness (see supplement S1). These algorithms are stored on the camera's SD card, and the AI module runs inference on four images captured by the image sensor. Once the system identifies a likely object of interest (greater than a certain probability to remove false positives such as moving vegetation), it transmits photo notifications directly to officials or researchers via the cellular network through email or messaging applications (e.g., Telegram, WhatsApp). Where the cellular network is available, the time from detection by the motion sensor to the image appearing in a cell phone app or email can be very fast (less than a minute). This speed of transmission permits park managers the chance for rapid response to intrusion by poachers or to alert villagers to the nearby presence of tigers. For camera-alert systems deployed outside of the range of a cellular data network, the system also has the capability of relaying the notification from the field unit to the cellular network through an intermediating long-range radio transmitter (e.g., IRNAS 2018, Zualkernan et al. 2022, Whytock et al. 2023) or with the addition of a hop or repeater unit and then on to the end user via a satellite connection. Transmission using the long-range radio protocol is on the order of 3–10 minutes (the noncellular system is not covered in the present article; see supplement S1 for more details on long-range radio communications).

Because most trigger events are false positives, AI on-the-edge camera systems are able to filter out nontarget species and false triggers and send only a fraction of the images captured by the camera. TrailGuard AI innovations result in dramatic savings in battery life and, therefore, more transmissions of images on the same battery charge in the following ways: First, because the transmission of images is many times more costly than loading and running inference using the AI module—a step measured in hundreds of milliseconds—filtering using embedded AI saves on transmission of many unwanted images. Second, when it is not in action, the entire system is powered off and draws only 7–10 microamps (quiescent current; see supplement S1). Therefore, where a cell connection is available, this AI system can transmit on a single charge an average of about 2300 jpeg images, text files (containing metadata on battery levels, probability value of detected object, etc.) and still allow for about 1500–2000 false trigger events recorded to the SD card but not transmitted. Filtering images on the edge also reduces overloading the party receiving notifications with false alarms that, if they are allowed to progress, could habituate the end user to ignore them. Once a detection alert is received by a ranger or central command center, on the basis of the photo and the specific camera location, our working hypothesis is that staff can determine whether there is indeed a potential poacher or an animal that may initiate conflict and can respond accordingly. A host of options then become available, including communicating with villagers about the animal's presence to allow them time to guard livestock or avoid the location and sending a rapid response team to prevent the killing of tigers or other wildlife. In addition, including village leaders on the notifications empowers them to warn of a potential conflict situation.

**Figure 2.**
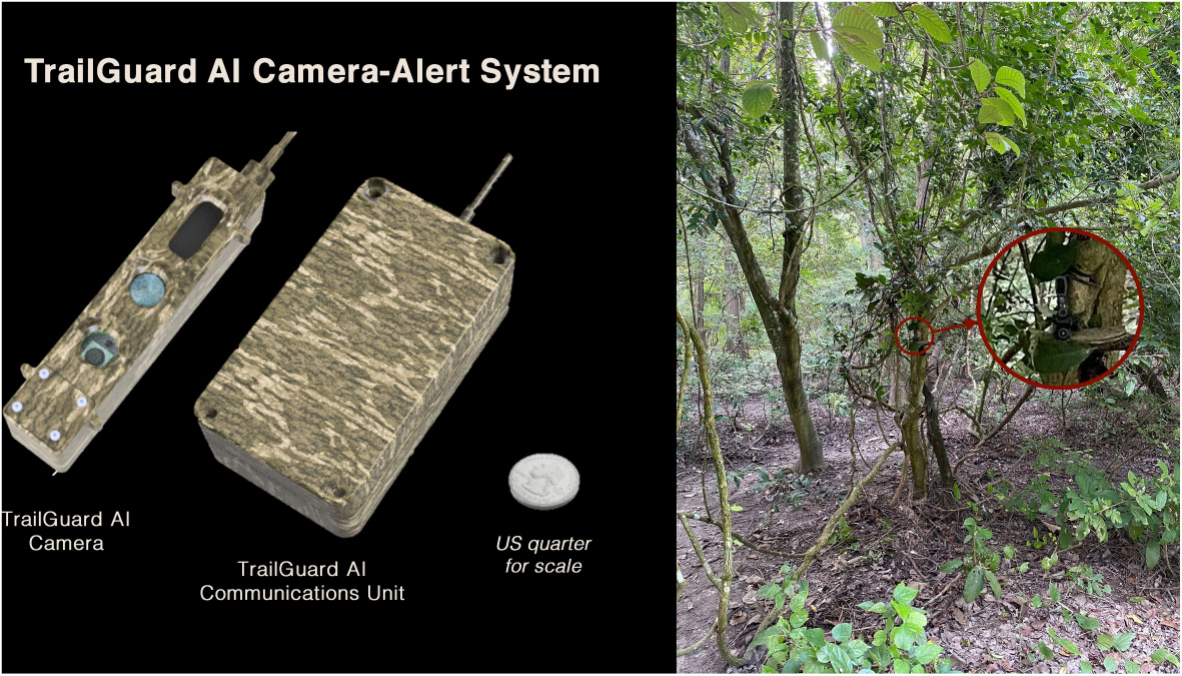
The TrailGuard AI camera system (left, US quarter for scale) can be well camouflaged near a trail such as this one in Dudhwa Tiger Reserve, as seen from the trail, that sent multiple real-time notifications of poachers without being detected (right).

The camera part of the alert system is much smaller than typical camera traps—about the size of a large Sharpie pen (with a length of 138 millimeters [mm], a width of 14 mm, and a depth of 11 mm for the version used in this study)—and is wired to a separate communications device (about the size of a small notepad; see supplement S1). This small form factor allows for easier camouflage of the camera, and the communication device can be placed out of site higher on a tree or behind the tree. The camera-alert system is also ideally positioned several meters from the trail and placed above eyeline, pointed at a downward angle toward the trail (figure 2; supplemental figure S1) in contrast to traditional camera installations that are often much more difficult to camouflage right next to trails near ground level. The camera's small size and camouflage capabilities are advantageous for effective security of the device; its relative secrecy is an important component for conflict resolution. Another important component is having a system that is easy to operate; in this case, deployment requires only connecting the battery and securing the camera to a tree.

We deployed TrailGuard AI for the first time in India in the vital Kanha–Pench corridor of central India with the objectives of transmitting the first tiger images and piloting education modules for forest department staff and local community members. This pilot deployment is embedded in a larger study of the social and cultural underpinnings of human–wildlife conflict in tiger landscapes, for which we (HN) conducted 644 total survey-based interviews and an additional 260 in-depth qualitative interviews. Twenty different villages and 10 castes were included, and although they had general attitudes toward technology, they were not yet familiar with AI-embedded detection systems. We then conducted another deployment within Dudhwa Tiger Reserve, in northern India, with the objective of examining the camera's use in rapidly notifying forest rangers of potential poachers and tigers occupying sections of buffer zones close to villages where conflict with humans was of grave concern to park authorities. Since this initial deployment, described in the present article, TrailGuard AI has continued to transmit data from the Kanha–Pench landscape and several other tiger reserves in India and Nepal and in parks in Africa and Central America (see supplement S1). The results in the present article are limited to a shorter-term deployment in Kanha–Pench and Dudhwa Tiger Reserve (Terai-Arc Landscape).

## TrailGuard AI deployment and capacity building

The Kanha–Pench corridor of central India covers an approximately 3150 square kilometer area and provides a sinuous approximately 140-kilometer link of forests between Pench Tiger Reserve to the southwest and Kanha Tiger Reserve to the northeast (figure 1b). The connection is vital to maintain the Kanha–Pench–Achanakmar population block, which, at over 300 tigers, is the largest tiger population in central India (table 1) and, therefore, one of the most important tiger populations in the world (Jhala et al. 2019). This link for tiger movement also contains hundreds of villages within or directly adjacent to the corridor, with more than 2.7 million people living within a 5-kilometer buffer of the full Kanha and Pench TCLs. The confluence of rural villagers and dispersing or resident tigers results in hundreds of livestock in the corridor being killed annually by tigers and the threat of retaliatory killing or poaching of tigers by people (Yumnam et al. 2014). Demonstrating how to deploy real-time camera-based alert systems in this vital and complex ecosystem would provide lessons for most other TCLs.

Dudhwa Tiger Reserve is a collection of protected areas—specifically, Dudhwa National Park and Kishanpur and Katarniaghat Wildlife Sanctuaries, in the northern India state of Uttar Pradesh along the Nepal border (figure 1a). The approximately 1310 square kilometers of protected lands and surrounding landscape contains approximately 107 individual tigers as well as the endangered greater one-horned rhinoceros (*Rhinoceros unicornis*), and Asian elephants (*Elephas maximus*; Jhala et al. 2019). Human densities are especially high surrounding Dudhwa and the poaching threat to tigers and the highly dense prey populations is of particular concern (Jhala et al. 2019, Wong and Krishnasamy 2022).

We deployed 12 TrailGuard AI camera-alert systems simultaneously from mid-May to mid-July 2022 for a total of 591 trap nights in the Kanha–Pench corridor and seven camera-alert systems in the Dudhwa Tiger Reserve in early September 2022 to mid-December for approximately 705 trap days (see supplemental file S2 for an explanation). Camera-alert systems in the Kanha–Pench corridor were deployed in nine contiguous forest department beats (i.e., a beat is considered the smallest administrative unit) with some of the highest spatial densities of reported depredations of livestock by tigers in the corridor. We collaborated with local rangers and guards to identify crossover areas with recent tiger activity and cell data coverage. All the TrailGuard AI locations were within one kilometer of the forest–agriculture interface primarily on trails and roads that led directly into villages. In addition, one system was positioned near a recent livestock kill to test the use case of monitoring for people potentially poisoning carcasses.

**Figure 3.**
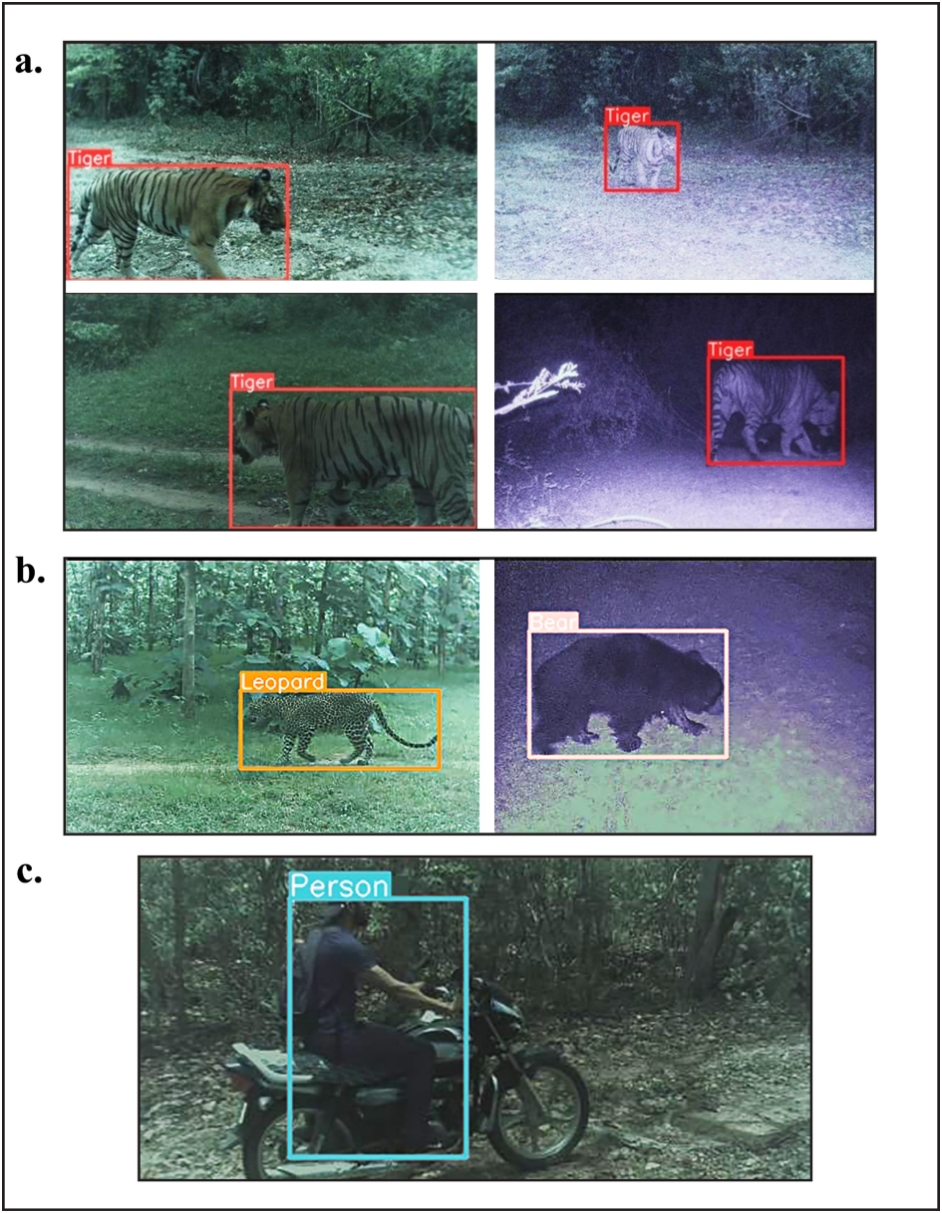
Images captured by TrailGuard AI and then transmitted via the cellular network as (a) tiger detection notifications. The tiger edge detector was extremely accurate, with a median probability value of .9883. We also captured and transmitted detections of (b) other conflict-prone species, including leopard and sloth bear, by setting the detection probability for an animal species to be very low, turning, in this case, the detector targeting tigers into a megadetector (see the supplemental material). Images of humans were also successfully transmitted from a camera location where (c) cell connectivity was absent, instead relying on long range radio and a range-extending repeater unit to a gateway within range of a cell tower.

All TrailGuard AI units transmitted images in the Kanha–Pench corridor study, 8 of 12 detected and transmitted images of tigers (figure 3). The TrailGuard AI system detected tigers in 61 trigger events with high accuracy: The median probability value for the tiger detector running on the edge was *p* = .9883 (supplemental table S2).

Researchers and forest department officials received notifications of tiger presence via email or push notification between 30 and 42 seconds after detections. These notifications constitute the first ever transmission of wild tiger detections using embedded AI. These notifications included tiger detections from three units that were within 300 meters of a village and from which there were also daily notifications of villagers grazing cattle or collecting forest products. In addition, a tiger was detected revisiting a recent livestock kill, feeding, and subsequently moving the carcass. Besides tigers, we also received rapid notifications during the deployment period of other conflict-prone species including leopard (*Panthera pardus*), sloth bear (*Melursus ursinus*), and wild boar (*Sus scrofa*; see supplement S1). Forest department officials kept the village leaders informed via text chat of any detections they deemed of particular concern, although, in this case, not in a rapid manner, and our sample size was too low to determine any influence on instances of conflict.

**Figure 4.**
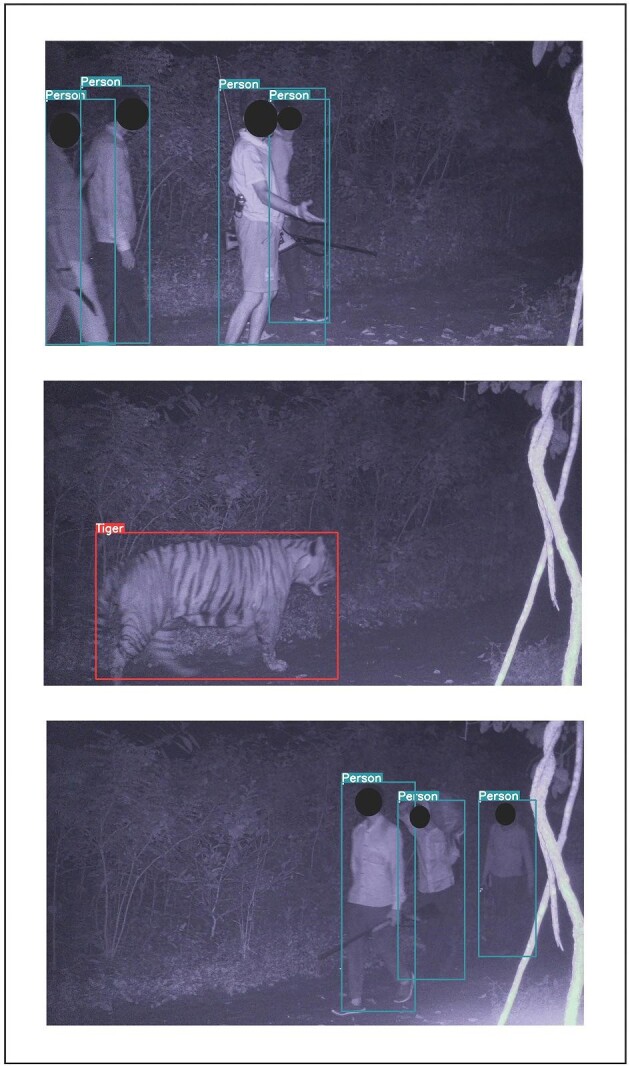
Detections of poachers and a tiger in Dudhwa Tiger Reserve, India. A TrailGuard AI camera detected the same gang of poachers on two different nights, including an image of one poacher carrying a dead chital (the lowest image). Authorities identified the individuals from the real-time image notifications. Tiger detections were also transmitted from the same location between the poacher detections.

All seven of the units deployed in the Dudhwa Tiger Reserve successfully transmitted real-time images, but only three during this study period transmitted objects of interest from within the tiger reserve to forest department officials. One unit within Kishanpur Wildlife Sanctuary captured a group of poachers carrying a gun and knives on two separate dates, and a presumably poached chital (*Axis axis*) on the second detection (figure 4). Authorities were able to quickly identify the individuals via the transmitted images and were investigating the matter to obtain more information on the alleged poachers’ current whereabouts. Tigers were detected in the same location 3 days before the second poacher detection, indicating the clear and present threat of poachers to endangered wildlife populations. Tiger detections are now being received by forest staff and researchers on a daily basis from both pilot deployment areas, with greater than 70 unique tiger notifications having been transmitted and received. Applicable data is provided in the figshare.org online repository. (For more information on detection of poachers, see supplement S1.)

## Education modules

Successful deployment of this technology will be aided by the adoption by field personnel and, depending on the scenario, assistance by local community members. We conducted two different education modules for the forest department divisions of the Kanha–Pench corridor and one module for villagers. The forest department module introduced broader issues of human–wildlife conflict and wildlife connectivity before introducing the use and installation of TrailGuard AI. Following the presentation of the module's elements, all 72 attendees were provided with open-ended discussion questions concerning the actions they might take if they received a camera notification, how they would deploy the camera system to address human–wildlife conflict, and how they would effectively incorporate the village communities when responding to camera-system notifications. All staff members in attendance contributed their insights and provided an extensive response on specific human–wildlife conflict scenarios in each of their administrative units, as well as their thoughts on how the camera system would be effective in dealing with such instances. Some notable responses include the camera system's utility in preventing the poisoning of livestock carcasses and waterholes, raising villagers’ awareness of tiger presence quickly, dispersing mobs before they become out of control and attempt to harm the predator, and monitoring livestock grazing in areas in which tigers are active. Furthermore, there was agreement that the camera-alert system could “help fill the gaps in staff presence, particularly in high-conflict areas” (i.e., serving as a force multiplier for overstretched staff). Although the cell coverage proved adequate for our deployments, the staff indicated that the system would need to operate mostly in areas with no cell data coverage (see supplement S1).

The purpose of the module for villagers was to educate and produce a dialogue with local community members directly affected by tiger conflict and to identify a community steward to employ to assist with maintenance of the camera-alert system. This module presented a general framework of stewardship and engagement to strengthen community capacity, develop understanding in efforts toward mitigating human–carnivore conflict through targeted education material, foster stewardship and empowerment through local community participation. The elements of the module included information on the cultural significance of tigers, the ecological importance of the species for the health of the forests in which the villagers subsist, and the global threats facing tiger populations. The module then discussed the function of TrailGuard AI and the aim to assist in reducing instances of conflict. The workshop was held in one of the villages near where the camera was installed, in the presence of local forest department personnel. Twenty-six village members attended. We prepared general questions for the villagers, including their concerns about the system being installed in nearby forests, actions they might take if they received news of an approaching tiger, and how they thought tiger detections could best be used to support village members. The villagers, primarily the men, had many questions about the technology and had several suggestions about how the camera system could reduce the present high depredation incidences. The village head informed us about their negative experiences with other wildlife species, such as leopards, and was curious to learn whether the camera could serve as a warning system for leopards and other problematic species. The dialogue with the villagers was documented by the team. The participants in this workshop were optimistic about the camera system and expressed a willingness to participate in related workshops and stewardship initiatives.

## A transformative shift in the use of camera data for conservation

The use of real-time systems such as TrailGuard AI is a major shift from the generally passive use of camera trap data to the active use of AI-filtered camera trap notifications. Compared with camera trap studies in which the design, collection, and analysis of photos can take place siloed within a research group or management unit, the use of these systems’ full potential requires the active involvement of field staff that have day-to-day interactions with the poaching threat and human–wildlife conflict. There must be buy-in from these personnel because the utility of real-time data for prevention of poaching or conflict could be lost without rapid action by the proper authorities. Engaged collaborations will be important to assist with initial training on the deployment of the camera system and lessons learned in using the data notifications.

Real-time camera data also opens a new realm of socioecological research questions, whose answers will aid the efficacy of conservation efforts using these technologies. These questions could include when and where poaching incidents decrease after system deployment, an evaluation of timely actions by resource managers related to detection notifications, and the subsequent response of local residents to intelligence about the presence of conflict-prone species. In areas close to human habitation where the goal is the prevention of human–wildlife conflict, further research and management actions should be coupled with initiatives that educate people on the importance of wild tigers and promote equitable cooperation between local communities and frontline staff (Johnson et al. 2018, Ardoin et al. 2020).

**Figure 5.**
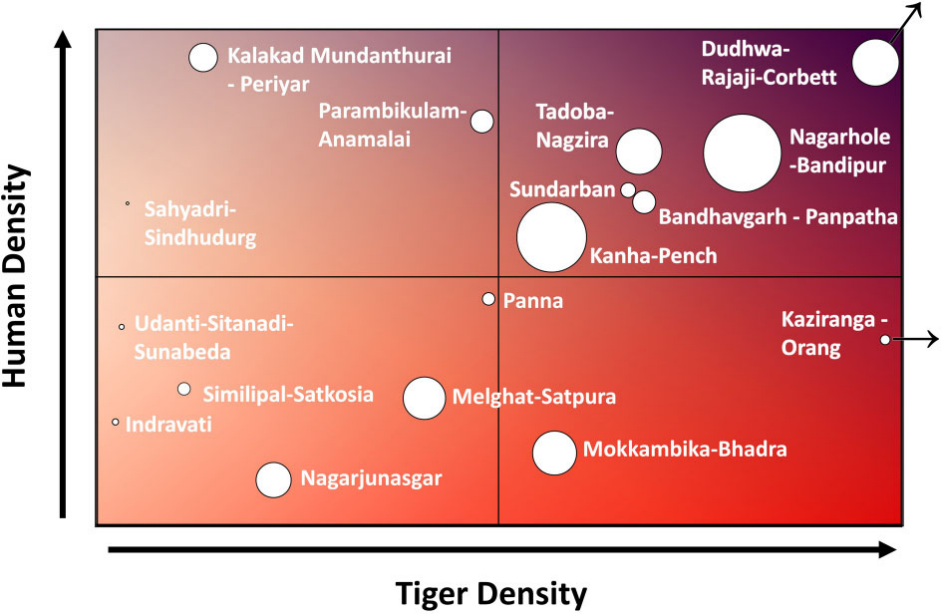
Conceptual matrix of where real-time camera deployments directed toward abating human–tiger conflict or for stopping poaching may be most appropriate. Strategies for only preventing poaching would be appropriate with increasing tiger density but low human density (the lower quadrants), whereas with increasing human density (the upper-right quadrant), camera deployments may shift to preventing more human–tiger conflict and poaching. With most of the tiger populations of India applied to the matrix, it is notable that some of the largest and densest tiger populations are also in areas with relatively high human density, indicating camera deployment to prevent conflict and poaching would be most appropriate. The circle size is scaled by land area, and the Dudhwa–Rajaji–Corbett and Kaziranga–Orang populations are outliers that could extend further beyond the figure boundaries relative to the other tiger populations.

Adaptability is necessary for the deployment of these systems, depending on the situation and threat. We plotted the relationship of tiger densities to human densities across India's TCLs as a conceptual model to illustrate where differing deployment methodologies may be considered with lowest density both of humans and of tigers in the lower-left corner, moving to the highest densities of both in the upper right (figure 5). Within parks and protected areas where there is a lower density of people and an increasing density of tigers (lower two quadrants), the poaching threat could be more pronounced. In this circumstance, the discretion of camera sites and the camouflage of cameras are necessary to detect intruders involved in poaching or illegal logging or engaged in other illicit behavior (figure 5). Poachers will likely avoid a trail or general area if they have learned of the TrailGuard AI locations; therefore, stealth is necessary. In these cases, capacity-building modules on system deployment would only be appropriate for forest department staff and not for community members, because widespread knowledge of TrailGuard AI locations could be counterproductive to an antipoaching campaign. As was noted, systems such as TrailGuard AI are advantageous because the unit itself is much smaller than typical camera traps and can be hidden away well above the eyeline, and the AI models (CVEDIA) are agnostic to camera angle, working accurately at 5 meters above the ground for human detection and for key wildlife species (see supplement S1).

Alternatively, in areas in which human–wildlife conflict is prevalent, hiding camera-alert system locations may become difficult because of high human density and the need to relay detection information to residents to avoid conflicts from occurring (see the upper-right quadrant of figure 5). This is especially true for some of the most important tiger landscapes in the world, including Nagarhole–Bandipur, Dudhwa–Rajaji–Corbett, and Kanha–Pench, large landscapes that contain some of the highest human and tiger population densities. Not informing local residents at least of the general deployment raises ethical questions (typically ignored by researchers; see Sharma et al. 2020) and may be counterproductive if the goal is to prevent livestock being killed, crops being raided (e.g., by elephants), or people being attacked. An approach in which local communities are involved, well informed, and empowered, such as the inclusion of a paid community steward for camera security and maintenance, can increase community support. This can increase trust, which aids in efforts for community member action when given important information about potential threats, ultimately reducing human–wildlife conflict. This is in contrast to well intentioned but poorly designed efforts with limited inclusion of local stakeholders, which can reduce their receptivity to action and commitment to conservation goals (Reed 2008, Leong et al. 2009).

India and Nepal have some of the highest human densities surrounding large tiger populations, making them some of the best test sites to improve deployment methods and community involvement initiatives (figure 5). In addition, there is potential for movement between populations creating a greater metapopulation, the largest example of which is in central India and includes the tiger populations of Bandhavgarh–Panpatha, Kanha–Pench, Melghat–Satpura, Panna, and Tadoba–Nagzira (figure 5). As movement among these populations increases, continued studies of techniques to best maximize deployment of alert systems far outside protected areas will be key to the reduction of human–tiger conflict.

In a recent paper, Whytock and colleagues (2023), working in a tropical forest environment in Africa, showed that it is possible to send reliable, real-time information from camera traps over a satellite network (Iridium) by integrating an artificial intelligence model, off-the-shelf camera traps, and custom hardware. Our solution using TrailGuard AI also transmits over cellular, long-range radio, and a satellite network (Inmarsat), with the additional capability of also sending jpeg images in real-time. Whytock and colleagues (2023) also suggested that scaling up a camera-alerting system will require engineering expertise and, potentially, commercial production and hoped that the broader solutions they presented in their paper can inform future efforts to successfully design and deploy robust, connected camera traps that provide real-time insights for conservation and ecology. TrailGuard AI has begun to set the stage for addressing all three of these goals by bringing together the expertise of hardware, software, and communications engineers from Intel, ANFA engineering, VVDN, CVEDIA, and Inmarsat, working with engineers from RESOLVE to design an efficient alerting system for widespread use that can transmit data from anywhere; by producing units commercially and soon at a scale to meet the demand; and by creating a system designed and informed by wildlife biologists for specific use by biologists and wildlife managers rather than adapting systems designed for the hunters’ market.

Technologies such as TrailGuard AI have the potential to be important tools for conserving tigers and many other apex predators, both inside and outside areas of human habitation. We have shown that this technology can work to detect and transmit tiger and human images in less than a minute and that a regional group of on-the-ground personnel acknowledge its potential to aid in conflict and poaching abatement. Once sufficient images of tigers are generated in a landscape, it will be possible to add reidentification algorithms on the server to alert park managers to the recurring presence of problem animals and improve response time. Similarly, the next phase of TrailGuard AI deployment will start incorporating new technology that links the edge detection of a conflict-prone species such as tigers with deterrence technology, the ultimate goal.

Finally, the application of systems such as TrailGuard AI goes well beyond the conservation of tigers to other imperiled large mammal species for which AI detector models have been developed by CVEDIA (see supplement S1), such as snow leopards (*Panthera uncia*), Asiatic elephants, various species of bears, and gray wolves (*Canis lupus*). The camera-alert system has shown its utility in protecting African savanna elephants (*Loxodonta africana*) by alerting rangers and the rangers subsequently apprehending multiple groups of poachers in Serengeti National Park and other protected areas of eastern Africa (Dinerstein 2018). Used correctly and at the appropriate density for the unique situation, this camera-alert system has the potential to improve direct, timely intervention for wildlife conservation.

## Supplementary Material

biad076_Supplemental_File

## Author Biography

Jeremy Dertien (jdertien1@gmail.com) is a postdoctoral researcher, Hrishita Negi is a PhD candidate, and Robert Baldwin is a professor and endowed chair at Clemson University, in Clemson, South Carolina, United States. Eric Dinerstein (edinerstein@resolve.ngo) is the director of Nightjar and is a senior biodiversity advisor at RESOLVE, Piyush Yadav is a conservation technology fellow at RESOLVE, Andy Lee is a research associate at RESOLVE and a program coordinator of Nightjar, and Steve Gulick is the inventor of TrailGuard AI and an adviser to RESOLVE, in Washington, DC, United States. Ramesh Krishnamurthy is a senior scientist at the Wildlife Institute of India, in Dehradun, Uttarakhand, India. Rajesh Gopal is the secretary general, H. S. Negi is a senior advisor, and Mohnish Kapoor is a project coordinator for the Global Tiger Forum, in New Delhi, India. Mijail Benitez is a vice president, AJ Wijnveen is chief scientist and CEO, and Miguel Ferreira, is a lead computer scientist at CVEDIA Ltd, in Reading, Berkshire, United Kingdom. Sanjay Kumar Pathak is the field director of Dudhwa Tiger Reserve, in Uttar Pradesh, India. Brett Wright is the director of Tigers United University Consortium and a professor at Clemson University, in Clemson, South Carolina, United States.

## References

[bib1] Appleton MR , et al. 2022. Protected area personnel and ranger numbers are insufficient to deliver global expectations. Nature Sustainability 5: 1100–1110.

[bib2] Ardoin NM , Bowers AW, Gaillard E. 2020. Environmental education outcomes for conservation: A systematic review. Biological Conservation 241: 108224.

[bib3] Carter NH , Linnell JDC. 2016. Co-adaptation is key to coexisting with large carnivores. Trends in Ecology and Evolution 31: 575–578. 27377600 10.1016/j.tree.2016.05.006

[bib4] Dhanwatey HS , Crawford JC, Abade LAS, Dhanwatey PH, Nielsen CK, Sillero-Zubiri C. 2013. Large carnivore attacks on humans in central India: A case study from the Tadoba–Andhari Tiger Reserve. Oryx 47: 221–227.

[bib5] Dinerstein E. 2018. Fighting Illegal Poaching with a Purpose-Built AI Camera. Intel Corp.

[bib6] Dinerstein E , et al. 2006. Setting Priorities for the Conservation and Recovery of Wild Tigers 2005–2015. Save the Tiger Fund.

[bib7] Dinerstein E , et al. 2007. The fate of wild tigers. BioScience 57: 508–514.

[bib33] Global Forest Watch . 2022. Global Forest Watch Open Data Portal. Global Forest Watch. https://data.globalforestwatch.org.

[bib8] Global Tiger Forum . 2019. Action Tiger: Tiger Action Plans of 13 Tiger Range Countries. Global Tiger Forum.

[bib9] Global Tiger Initiative Secretariat . 2011. Global Tiger Recovery Program 2010–2022. World Bank.

[bib10] Glover-Kapfer P , Soto-Navarro CA, Wearn OR. 2019. Camera-trapping version 3.0: Current constraints and future priorities for development. Remote Sensing in Ecology and Conservation 5: 209–223.

[bib12] Goodrich JM. 2010. Human–tiger conflict: A review and call for comprehensive plans. Integrative Zoology 5: 300–312. 21392348 10.1111/j.1749-4877.2010.00218.x

[bib11] Goodrich J , Sanderson AJ, Chapman E, Gray S, Chanchani TNE, Harihar P. 2022. Tiger: Panthera tigris. The IUCN Red List, International Union for Conservation of Nature. www.iucnredlist.org/species/15955/214862019.

[bib11a] Hahn NR , Bombaci SP, Wittemyer G. 2022. Identifying conservation technology needs, barriers, and opportunities. Scientific Reports 12: 4802. 10.1038/s41598-022-08330-w. 35314713 PMC8938523

[bib13] Inskip C , Zimmermann A. 2009. Human–felid conflict: A review of patterns and priorities worldwide. Oryx 43: 18–34.

[bib14] [IRNAS] Institute for Development of Advanced Applied Systems . 2018. Animal Conservation with LoRaWAN: Turtles, fish, and more. IRNAS. www.irnas.eu/animal-conservation-with-lorawan-turtles-fish-and-more.

[bib16] Jhala YV , Qureshi Q, Nayak AK. 2019. Status of Tigers, Copredators and Prey in India: 2018. National Tiger Conservation Authority, Government of India, and Wildlife Institute of India, Dehradun. Technical report no. 2019/05.

[bib15] Jhala YV , Gopal R, Mathur V, Ghosh P, Negi HS, Narain S, Yadav SP, Malik A, Garawad R, Qureshi Q. 2021. Recovery of tigers in India: Critical introspection and potential lessons. People and Nature 3: 281–293.

[bib17] Johnson MF , Karanth KK, Weinthal E. 2018. Compensation as a policy for mitigating human–wildlife conflict around four protected areas in Rajasthan, India. Conservation and Society 16: 305–319.

[bib18] Joshi AR , et al. 2016. Tracking changes and preventing loss in critical tiger habitat. Science Advances 2: 1501675. 10.1126/sciadv.1501675 PMC4820387 27051881

[bib19] Karanth KK , Naughton-Treves L, Defries R, Gopalaswamy AM. 2013. Living with wildlife and mitigating conflicts around three Indian protected areas. Environmental Management 52: 1320–1332. 24026255 10.1007/s00267-013-0162-1

[bib20] Karanth KU , Nichols JD, Harihar A, Miquelle DG, Samba Kumar N, Dorazio RM. 2017. Field practices: Assessing tiger population dynamics using photographic captures. Pages 191–224 in Karanth KU, Nichols JD, eds. Methods For Monitoring Tiger and Prey Populations. Springer.

[bib21] Kays R , et al. 2022. SNAPSHOT USA 2020: A second coordinated national camera trap survey of the United States during the COVID-19 pandemic. Ecology 103: e3775. 35661139 10.1002/ecy.3775 PMC9347782

[bib22] Leong KM , Forester JF, Decker DJ. 2009. Moving public participation beyond compliance: Uncommon approaches to finding common ground. George Wright Forum 26: 23–39.

[bib23] Letro L , Fischer K. 2020. Livestock depredation by tigers and people's perception towards conservation in a biological corridor of Bhutan and its conservation implications. Wildlife Research 47: 309–316.

[bib24] Milda D , Ramesh T, Kalle R, Gayathri V, Thanikodi M. 2020. Ranger survey reveals conservation issues across protected and outside protected areas in southern India. Global Ecology and Conservation 24: e01256.

[bib25] Miller JRB , Jhala Y V., Jena J. 2016. Livestock losses and hotspots of attack from tigers and leopards in Kanha Tiger Reserve, Regional Environmental Change 16: 17–29.

[bib26] Reed MS. 2008. Stakeholder participation for environmental management: A literature review. Biological Conservation 141: 2417–2431.

[bib27] Rich LN , Miller DAW, Robinson HS, McNutt JW, Kelly MJ. 2016. Using camera trapping and hierarchical occupancy modelling to evaluate the spatial ecology of an African mammal community. Journal of Applied Ecology 53: 1225–1235.

[bib28] Sharma K , Fiechter M, George T, Young J, Alexander JS, Bijoor A, Suryawanshi K, Mishra C. 2020. Conservation and people: Towards an ethical code of conduct for the use of camera traps in wildlife research. Ecological Solutions and Evidence 1: e12033.

[bib29] Sunquist M , Sunquist F. 2002. Wild Cats of the World. University of Chicago Press.

[bib30] Whytock RC , et al. 2023. Real-time alerts from AI-enabled camera traps using the Iridium satellite network: A case-study in Gabon. Methods in Ecology and Evolution 14: 867–874.

[bib31] Wikramanayake E , et al. 2011. A landscape-based conservation strategy to double the wild tiger population. Conservation Letters 4: 219–227.

[bib32] Wong R , Krishnasamy K. 2022. Skin and Bones: Tiger Trafficking Analysis from January 2000–June 2022. TRAFFIC, Southeast Asia Regional Office.

[bib32a] WorldPop . 2018. Unconstrained Global Mosaics. Global High Resolution Population Denominators Project. 10.5258/SOTON/WP00647.

[bib34] Yumnam B , Jhala YV, Qureshi Q, Maldonado JE, Gopal R, Saini S, Srinivas Y, Fleischer RC. 2014. Prioritizing tiger conservation through landscape genetics and habitat linkages. PLOS ONE 9: e111207. 25393234 10.1371/journal.pone.0111207 PMC4230928

[bib35] Zualkernan I , Dhou S, Judas J, Sajun AR, Gomez BR, Hussain LA. 2022. An IoT system using deep learning to classify camera trap images on the edge. Computers 11: 13.

