# Supplementary material for: Mitigating human–wildlife conflict and monitoring endangered tigers using a real-time camera-based alert system
Source: Bioscience. 2023 Sep 14;73(10):748–57. doi: 10.1093/biosci/biad076 (PMC10580963; doi:10.1093/biosci/biad076)
Supplement: biad076_Supplemental_File [file biad076_supplemental_file.pdf]

Table S1. Human population change estimated between the years 2000 and 2020 within a 5km buffer of the world’s Tiger Conservation Landscapes (TCL). Of the 76 TCLs, 69 showed positive human population growth within and surrounding the critical tiger habitat.

TCL ID#	TCL Name	20-year Population Change
1	Heilongjiang	-2,456
2	Russian Far East - China	-142,335
3	Bukit Barisan Selatan South	85,773
4	Bukit Balai Rejang - Selatan	59,031
5	Kerinci Seblat	651,387
6	Bukit Rimbang Baling	247,214
7	Bukit Tigapuluh Landscape	212,473
8	Tesso Nilo Landscape	116,939
9	Kualar Kampar-Kerumutan	278,437
10	Berbak	30,588
11	Bukit Barisan South	84,379
12	Rimbo Panti-Batang Gadis West	28,011
13	Sibologa	66,985
14	Gunug Leuser	347,027
15	Endau Rompin	1,159
16	Taman Negara - Belum	1,893,050
17	Krau	20,942
18	Khlong Saeng	71,314
19	Tenasserims	-194,348
20	Salak-Phra	3,131
21	Phu Miang - Phu Thong	42,669
22	Phu Khieo	-54,390
23	Khao Yai	62,424
24	Thap Lan - Pang Sida	-2,386
25	Cardamom's	621,291
26	Cambodian Northern Plains	1,487,915
27	Southern Annamites	908,346
28	Cat Tien	202,172
29	Bi Dup-Nui Ba	34,854
30	Kon Ka Kinh	117,584
31	Yokdon	77,499
32	Xe Bang Nouan	12,603
33	Hin Nam Ho	22,059
34	Northern Annamites	291,607
35	Nam Et Phou Loey	273,752
36	Nam Ha	37,929
37	Northern Forest Complex - Namdapha - Royal Manas	1,813,724
38	Kaziranga - Garampani	500,024
39	Sundarbans	91,608
40	Chitwan	912,326

41	Bardia South	141,775
42	Bardia	506,814
43	Suklaphanta	1,220,988
44	Corbett - Sonanadi	1,069,209
45	Rajaji Minor	701,198
46	Rajaji Major	120,601
47	Panna East	122,824
48	Panna West	34,739
49	Bandhavgarh - Panpatha	216,568
50	Kanha – Phen	619,620
51	Pachmarhi - Satpura - Bori	264,580
52	Melghat	57,116
53	Pench	178,704
54	Andhari - Tadoba	141,036
55	Indravati	1,042,078
56	Sunabeda-Udanti	143,350
57	Satkosia-Gorge	118,764
58	Simlipal	111,772
59	Palamau	382,039
60	Painganga	41,355
61	Nagarjunasagar South	30,284
62	Nagarjunasagar North	15,053
63	Shendurney	56,117
64	Periyar - Megamala	98,941
65	Anamalai-Parambikulam	217,709
66	Western Ghats: Bandipur - Khudrenukh – Bhadra	199,331
67	Biligiri Range	-1,344
68	Western Ghats - Sharavathi Valley	-10,751
69	Dandeli - Anshi	11,639
70	Dandeli North	3,610
71	Radhanagari	49,496
72	Chandoli	3,022
73	Mahabaleshwar Landscape - South	80,214
74	Purna	84,738
75	Mahabaleshwar Landscape - North	88,992
76	Shoolpaneswar	44,160
	Total 20-year Population Change	19,518,652

Table S2. Probability values for tiger detector running on the edge in TrailGuard AI camera-alert systems deployed in the K-P Corridor, MP India.

Camera ID and Event #	Edge Detector Probability
68692D Event: 346	0.9990
68692D Event: 370	0.9941
63D80D Event: 98	0.9004
68692D Event: 380	0.8633
686B75 Event: 253	0.9004
68692D Event: 462	0.9990
687959 Event: 488	0.9980
686ACD Event: 466	0.9971
63D7B5 Event: 316	0.2500
63D7B5 Event: 320	0.9302
63D7B5 Event: 321	0.5293
686ACD Event: 470	0.9902
63D7B5 Event: 328	0.9883
63D7B5 Event: 329	0.6167
63D7B5 Event: 330	0.9116
686ACD Event: 473	0.9990
63D7B5 Event: 334	0.9980
687959 Event: 680	0.9873
63D7B5 Event: 335	0.9990
63D7B5 Event: 336	0.4734
63D7B5 Event: 337	0.9990
63D7B5 Event: 338	0.9863
63D7B5 Event: 340	0.9790
63D7B5 Event: 341	1.0000
686ACD Event: 498	0.9980
63D7B5 Event: 346	0.9980
6543DD Event: 83	0.9590
686ACD Event: 499	0.9893
63D7B5 Event: 347	0.2639
686A89 Event: 44	0.9990
6543DD Event: 115	0.9990
63D7B5 Event: 352	0.4185
63D7B5 Event: 354	0.3508
686A89 Event: 55	0.3835
686A89 Event: 56	0.9590
63D7B5 Event: 356	0.9951
6543DD Event: 133	0.8906
63D7B5 Event: 371	0.3005
63D7B5 Event: 373	0.2952
686ACD Event: 633	0.9932
686ACD Event: 641	0.9883
686ACD Event: 647	1.0000
686ACD Event: 648	1.0000

686ACD Event: 650	1.0000
686ACD Event: 654	1.0000
686ACD Event: 655	1.0000
6543DD Event: 213	0.9961
687959 Event: 1448	0.9302
686ACD Event: 663	1.0000
6543DD Event: 232	0.8037
687959 Event: 1455	0.8018
686ACD Event: 676	1.0000
687959 Event: 1473	0.9990
686ACD Event: 728	0.9971
686ACD Event: 729	0.9971
63D7B5 Event: 436	0.5620
686ACD Event: 731	0.9883
686ACD Event: 735	0.9971
68692D Event: 433	0.2251
63D7B5 Event: 343	0.4146
6543DD Event: 132	0.7617
**Number of Events**	**61**
**Median**	**0.9883**
**Mean**	**0.8450**
**Minimum**	**0.2251**

a.

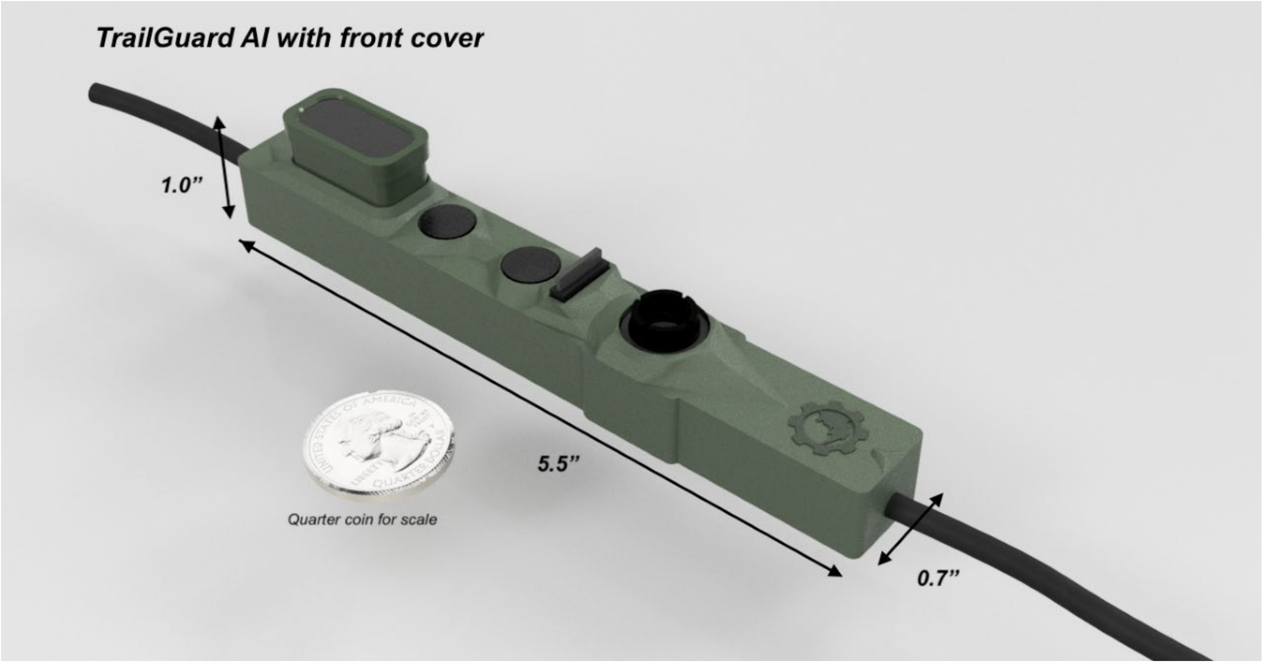

b.

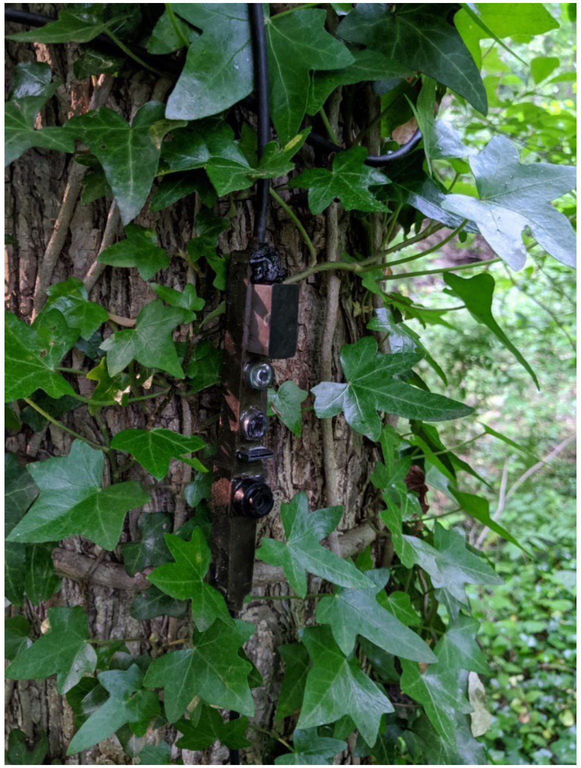

Figure S1. Images of the prototype TrailGuard AI. (**a**) The 3D rendering shows measurements in inches with a U.S. quarter coin for scale. (**b**) The camera can be affixed to a tree like a traditional camera trap or several meters above ground pointing at a downward angle towards a trail with great effectiveness.

Supplementary Materials

Technology overview of the real-time, camera-based alert system used in this study, TrailGuard AI

TrailGuard AI is an end-to-end, camera-based alert system designed for enhancing wildlife conservation and promoting human-wildlife coexistence in remote areas. The system can be deployed along trails or access points in areas of interest. TrailGuard AI autonomously detects target objects, and transmits real-time alerts that can trigger rapid-response to entry by poachers or illegal loggers, wildlife entering agricultural or pastoral lands, or status of endangered or exotic invasive species. TrailGuard AI is small and cryptic, allowing it to be easily camouflaged and to avoid detection, theft, or vandalism. Embedded AI in the camera automatically filters out false positives, thereby saving transmission costs and battery consumption; a single charge on the small battery can also operate the system for over 25 months in the field (when placed properly and restricted to the GSM version). The multipurpose communications unit allows transmission of alerts using GSM, LoRa, or satellite modem to designated authorities or researchers. The GSM version can transmit an image from the camera to authorized personnel in under 30 seconds. A variety of customizable AI detectors—including regional wildlife species, humans, logging trucks—can be interchanged in TrailGuard AI by simply swapping an SD card, thereby allowing the same hardware platform to be adopted for different use cases.

TrailGuard AI consists of the camera and core communications unit, connected by a 2-meter power/data cable (see Figure S1). In the field, when the PIR motion sensor is triggered by a passing object, the camera wakes up from a “Deep Sleep mode” to immediately capture four images. The core of the system is Intel’s low-power, high performance AI processor, the Myriad X chip. The Myriad X chip in the camera loads the AI algorithm in under 500 ms and runs inference on all four images, a process occurring in milliseconds, to determine if there is a positive detection of a target object (e.g., human, animal species). If there is no positive detection, the system writes all four images to the SD card and returns to “Deep Sleep mode”. If a true positive is detected, a single 20KB image will be transferred to the communications unit, which then will transmit the jpeg file as a TrailGuard AI alert to designated recipients.

(Note: the camera-alert system used in the period covered by this field study in India relied on the Intel Myriad 2 computer vision chip. It functions in much the same way as the Myriad X chip, but the Myriad 2 system has been superseded by the more powerful Myriad X processor, now standard in all TrailGuard AI units. Similarly, the communications unit now being used in the field in May 2023, and described below, is two generations more advanced than the unit used in the 2022 field study in India).

Durability

Aside from the small form factor of the camera-alert system to avoid detectability, a major concern of researchers is the durability of the system, especially when placed in extreme environments such as humid tropical forests. To safeguard the electronics in the camera, the circuit boards are covered in special coatings and then encapsulated in a waterproof urethane material after being placed in the bottom of the enclosure and the sealed with a top plate to make the system rated waterproof (IP67--the IP67 rating refers to dustproofing and waterproofing).

The camera-alert system used in the India field study was encapsulated in urethane as were all units used in the field to date, globally. The urethane cures to a hardness that makes the outer layer of the camera extremely robust to abrasion or accidental dropping of the camera. The new versions of the communications units also receive these coatings and are placed in an IP67-rated enclosure. Connecting the camera to the communications unit is a waterproof M12 connector. The insulated cable is encased in stainless-steel tubing (to prevent chewing of the cable by arboreal mammals). It, too, is covered by a water-repellent material for camouflage.

During the pilot, one camera was temporarily sidelined when a grey langur monkey chewed through an unprotected data cable. The cable was quickly replaced. More importantly, the system has been operating in areas for over one year in daily proximity to langur and rhesus macaque troops and loss of units to primates has not been observed again. However, the latest version of the system with the stainless steel tubing protects the cable's wires from arboreal rodents and primates.

Other Critical Features of the System

Enhancing connectivity, adding Embedded AI, and conservation of battery life are three interrelated and critical variables affecting performance of the system in remote areas along with ease of concealment. These features are treated below.

Connectivity Options

Many wildlife habitats or locations prone to human-wildlife conflicts are remote and often lack reliable cell connectivity. TrailGuard AI offers a suite of connectivity solutions that allows the system to be deployed anywhere and reliably transmit real-time alerts using GSM, long-range radio (LoRa), or via a satellite network.

In areas where cellular connectivity (2G, 3G, 4G, LTE) is present, TrailGuard AI can transmit alerts out-of-the-box using a GSM protocol and a universal SIM card. The alerts can be received by the TrailGuard Gateway deployed in a location within range of a GSM cell tower, and relayed to the server. In locations where cell service is out of range, TrailGuard AI's communications unit can transmit alerts using a LoRa protocol (for a distance of 10–30 km depending on terrain and vegetation). These LoRa transmissions can be received and forwarded by a hop unit (repeater) and on to a satellite modem, taking between 3-10 minutes.
For more detailed information and a field guide to connectivity options using LoRa and satellite offered by TrailGuard AI, please contact edinerstein@nightjar.tech.

Embedded AI and Two-Tiered Filtering

TrailGuard AI can run inference on captured images using embedded AI algorithms stored on the microSD card in the camera. The results from prototype cameras equipped with the Intel Myriad 2 computer vision chip were very encouraging, obtaining a median probability value of the tiger edge detector of 0.9883 (n = 61 trigger events; Table S2).

The new version of TrailGuard AI incorporates the more advanced Intel Myriad X chip, which

offers state-of-the-art computer vision processing and can run heavy AI models with multiple output classes on the edge in less than a second. In addition, the new camera will have much improved low light sensitivity of the image sensor, which should result in better performance and thus higher inference values at night when tigers and most mammals are most active.

In the new Myriad X version, depending on the use case and geographic region, users can select from a collection of AI detectors to use in their TrailGuard AI systems. A new detector in production will offer a multi-species output class that includes the following classes: human, elephants, bears, wild felids (tiger, snow leopard, lion etc.), wild canids (wolves, dhole, coyote, African wild dog, dingo, etc.) rhinos, wild pigs (wild boar, warthogs, etc), vehicles (including logging trucks), and an Other category (essentially all quadrupeds with p values below the threshold for the vertebrate output classes). Each output class has its own folder on the SD card and images of that species or taxon are "binned" in those folders. Thus, the end user can skip searching the entire microSD card for "tigers" if they wanted to retrieve just a single higher resolution image of a tiger stored on the microSD card. From this new multi-species output class detector, users will be able to receive alerts from only those output classes of interest to them.

The AI detectors running on TrailGuard AI are developed by CVEDIA, a leading expert in the field of AI computer vision. CVEDIA's AI detectors are trained on synthetic data using 3-D renderings of target species and objects created by artists. These 3-D models are then placed in a variety of background environments, postures, positions, angles, lighting conditions, and level of occlusion in order to train the AI models to accurately identify the target and become agnostic to other environmental factors. This process gives CVEDIA's AI model advantages over traditional AI models trained on field data which can be biased towards particular conditions or simply limited by data availability for training models, particularly for rare wildlife species. Instead of needing a large volume of images for a rare species for which there is very limited camera trap data, the CVEDIA synthetic data approach only needs a small subset of images from a target species for model validation rather than training.

TrailGuard AI employs a two-tiered AI filtering technique to reduce false positive notifications before an alert reaches the Inbox of the end-user. Captured images are first filtered by an embedded AI algorithm in the camera. Additional filtering is performed on the server using a heavier-weight detector trained in Yolo v5 to detect wildlife species, humans, and vehicles, where there is no power constraint. Only then will the filtered images with positive inference be transmitted to users as notifications.

To further reduce overload of images at a Park Headquarters, selected individuals can receive TrailGuard AI image alerts as push notifications in a Telegram app on a smart phone. These alerts in Telegram arrive even faster than as an email attachment.

By changing settings in the software, or by running CVEDIA's People/Vehicle/Animal Detector in TrailGuard AI, the system functions as an edge-based version of Mega-Detector For more information on use of algorithms with TrailGuard AI, please contact edinerstein@nightjar.tech.

Battery Life and Power Consumption

Reducing power consumption and saving on battery life are essential to camera-alert systems deployed in remote areas. TrailGuard AI combines innovative engineering and AI to minimize battery usage, which significantly extends the system's operational life in the field, reduces resources required to upkeep monitoring, and avoids unnecessary disturbance to the deployment that could compromise concealment.

TrailGuard AI deployed along a trail stays in deep sleep mode with the entire system powered off, drawing only 7-10 micro amps (quiescent current) to keep a few vital sensors in alert state. Only when the passive infra-red motion sensor is triggered does the camera wake up and capture four images, the first in approximately 236 ms. Simultaneously, the onboard Myriad X computer vision chip, which draws very little power, loads a 13 MB CNN in about 500 ms and runs inference to determine if a target animal or intruder is detected. If the AI logic decides the trigger is from a false positive, the system will shut down and go back to sleep in about one second. If a true positive is detected, the image is transmitted to the communications unit and ready for a new trigger event in 2-3 seconds. This edge filtering of unwanted images using AI is a major power saver as transmission of images is many times more costly than loading and running inference for each trigger event. Similarly, the system is designed to save battery power using a number of other innovations in circuit board design, software advances, and choice of components. For example, by using custom software, it is possible to reduce transmission of a 20KB from trigger of motion sensor to appearing on the end user's cell phone to under 30 seconds. Most of this interval, about 20 seconds, is taken up by registration with the cell network. Other innovations reduce transmission time for LoRa and also feature other power savings protocols.

On a single charge, the 3.7v battery can transmit on average about 2,300 true positives over 4G/LTE. When TrailGuard AI is properly deployed to capture movement events of target animals and intruders, assuming two transmissions of true positives alert each day, it can last for 40 months in the field before the battery needs replacement or recharging. In contrast, all consumer trail cams with cellular connectivity (none offer LoRa connectivity) send every trigger event and have no edge filtering, thus potentially exhausting the battery in a matter of weeks to a few months.

Concealment and Deployment

Appropriate concealment is essential for the long-term operation of camera-alert systems in the field. For many traditional trail cameras, up to 40% of the units are reported lost due to theft or vandalism in the field after the first year of deployment. Proper concealment also helps with effective monitoring by avoiding raising suspicion by humans using the trails.

TrailGuard AI offers several features to improve concealment. First, the small form factor—for the camera used in this study were 138 mm x 14 mm x 11 mm—allows for easier camouflage of the camera while the communication device can be placed out of sight higher on a tree, or behind the tree. The camera and cable are meant to mimic a twig and a vine, respectively. Second, the ability to position the system high above ground level (~3 m) and pointed at a downward angle towards the trail, avoids detection from eye level (Figure S1). Performance of the AI algorithm detecting target objects is uncompromised by the elevated angle. Third, concealment is aided by use of an infrared illuminator instead of a white flash during nighttime image capture and using a

wavelength beyond the human threshold of detection. Finally, the ability to use materials from the local environment—such as detritus, bark, and elephant dung—to glue to the sides and front can further camouflage the unit.

To date, no TrailGuard AI unit deployed in the field has been lost to theft or vandalism. For more information about site selection and installation recommendations that can improve concealment of the system, please contact edinerstein@nightjar.tech.

Results-To-Date

The field deployment in and around four Indian tiger reserves covered here (Kanha, Pench, Kishanpur, Dudhwa) represent one example of the rollout of the technology globally. Since 2016, and increasing over the years, prototype versions of TrailGuard AI have been tested in the following regions and countries: Africa (South Africa, Zambia, Zimbabwe, Kenya, Tanzania); Europe (Germany, Italy, Ukraine); North America (Montana); Central America (Guatemala); and Asia (India, Nepal, and soon Indonesia (Sumatra)). Images have been transmitted successfully over various protocols. Poachers, illegal loggers, or intruders stealing park resources have been detected in 12 reserves to date, and multiple arrests in several results (Security-sensitive results cannot be shared here-please see www.nightjar.tech for more information). With TrailGuard AI featuring the Myriad X chip now in production, roll out to may more reserves are scheduled in 2023-2024.

Instructional Materials

To aid users before and during deployment of the technology and optimize its performance in a variety of environments, TrailGuard AI comes with a series of instructional materials available to end users and field deployment teams. These materials cover equipment setup and testing, site selection protocol, camouflaging the equipment, deployment tips, use of LoRa and a satellite network to enhance transmission, troubleshooting, and alert receipts and management etc. These following documents can be found at the website below; in the near future, some of these instructions will also be available in the form of short online videos.

- Quick Setup Guide
- Field Guide to Connectivity Options
- Do’s and Don’ts of TrailGuard AI Setup
- Wildlife Species Detectors

Website

Instructional materials, FAQs, and other related information about the TrailGuard AI technology can be found on this website: https://nightjar.tech/ and https://nightjar.tech/faq
